# p53 as a target in myeloproliferative neoplasms

**DOI:** 10.18632/oncotarget.719

**Published:** 2012-10-27

**Authors:** Min Lu, Ronald Hoffman

**Affiliations:** Division of Hematology/Oncology, Tisch Cancer Institute, Department of Medicine, Mount Sinai School of Medicine, New York, NY; Division of Hematology/Oncology, Tisch Cancer Institute, Department of Medicine, Mount Sinai School of Medicine, New York, NY

The Philadelphia chromosome negative myeloproliferative neoplasms (MPN) originate at the level of the pluripotent hematopoietic stem cell (HSC). JAK2V617F is present in virtually all patients with polycythemia vera (PV) as well as 50-60% of patients with primary myelofibrosis (PMF) and essential thrombocythemia (ET) [1]. Recently, Nakatake and coworkers demonstrated that JAK2V617F alters p53 responses to DNA damage by upregulating La antigen that increases MDM2 protein translation [2]. p53 plays a pivotal role in the maintenance of cell integrity in response to a variety of stresses by controlling target genes that regulate cell cycle arrest, apoptosis, senescence, DNA repair, or changes in metabolism. Based on these roles, p53 has been proposed as a promising molecular target for the treatment of a variety of cancers. Although about 50 % of cancers contain mutated forms of p53 which lead to loss of function, wild type p53 is universally present in PV, by contrast, p53 mutations have been identified exclusively in patients undergoing transformation to acute leukemia. The cellular levels of p53 are controlled by the rate at which it is degraded. MDM2 is the master regulator of p53, it regulates p53 levels through a negative feedback loop. MDM2 not only facilitates p53 degradation, but also binds p53 and inhibits its transcription. We have recently shown that MDM2 levels are increased in PV CD34^+^ cells while p53 mRNA levels are lower [3]. These observations lead to the exploration of therapeutic strategies to up-regulate p53 for the treatment of PV patients. Nutlins are small-molecule antagonists of MDM2, which specifically bind to MDM2, blocking MDM2-p53 interactions, resulting in p53 stabilization, accumulation and activation. This approach has been shown to inhibit tumor growth in a non-genotoxic manner in xenograft murine tumor models [4, 5]. MDM2 antagonists have the potential to be potent weapons to treat cancers containing wild type p53.

Recently, small-molecule inhibitors of JAK2 inhibitors have been shown to be effective in treating patients with advanced forms of myelofibrosis resulting in a reduction in the degree of splenomegaly and improvement in systemic symptoms but unfortunately the progeny of the malignant clone has not been documented to be substantially affected [6]. By contrast, interferon (IFNα) has been reported to reverse morphological marrow abnormalities, eliminate cytogenetic abnormalities, reduce or eliminate cells with JAK2V617F and result in the re-establishment of polyclonal hematopoiesis in selected patients with PV, essential thrombocythemia (ET) and early forms of primary myelofibrosis (PMF) [7]. We previously determined that IFNα specific targets PV JAK2V617F positive hematopoietic progenitor cells (HPC). IFNα activates a p38 mitogen-activated protein kinase (MAP kinase) resulting in apoptosis of PV HPC [8]. IFNα binds to the type I IFN receptor, and activates the JAK/TYK/STAT pathway, leading to multiple downstream events. Both the STAT1 and p38 MAPK pathways activate p53 [9]. Frequently protracted therapy of PV patients with IFNα is not possible due to a variety of adverse events necessitating its discontinuation. Since many of these adverse events are dose dependent, the identification of drugs which could be combined together with low doses of IFN would potentially provide a means of treating greater numbers of PV patients for longer periods of time.

We recently reported that combination treatment with sub-therapeutic doses of Peg IFNα 2a and Nutlin-3 significantly inhibited the proliferation and induced apoptosis in PV CD34^+^ cells as compared to each agent alone [3]. We also found that the combination of these agents at low doses decreased the proportion of JAK2V617F-positive HPCs. Both of these drugs affect p53 through two distinct pathways with Peg IFNα 2a activating p38 MAP kinase and STAT1 leading to increased p53 transcription and nutlin-3 prevents the degradation of p53 [3, 8]. These results strongly suggest that combinations of low doses of IFNα and nutlin-3 might serve as a novel therapeutic strategy for the long term treatment of PV patients.

RG7112 is a novel drug which acts as a selective inhibitor of p53-MDM2 binding and frees p53 from negative control, activating the p53 pathway in cancer cells. RG7112 is currently being evaluated in several clinical trials [10]. We predict that combination treatment with low doses of RG7112 or other second generation MDM2 antagonists will provide a promising strategy to treat a variety of blood cancers including PV.
